# The Rare Sore Throat: A Case of Thyroid Storm and Agranulocytosis

**DOI:** 10.5811/cpcem.6609

**Published:** 2023-12-06

**Authors:** Matthew J. Kunz, Wayne A. Martini, Levi Filler

**Affiliations:** * Creighton of Phoenix Arizona (COPA), Emergency Medicine, Phoenix, Arizona; † Mayo Clinic, Department of Emergency Medicine, Phoenix, Arizona; ‡ University of Arizona College of Medicine - Phoenix, Department of Emergency Medicine, Phoenix, Arizona

**Keywords:** case report, thyrotoxicosis, methimazole, bone marrow suppression, agranulocytosis

## Abstract

**Introduction:**

Thyroid storm is a rare but potentially life-threatening metabolic disorder that presents unique management challenges in the emergency department. Thionamides are commonly used as monotherapy for first-line treatment of hyperthyroidism.

**Case Report:**

In this case, a 26-year-old male presented to the emergency department with sore throat, fever, and diarrhea. He was found to have thyrotoxicosis as well as methimazole-induced bone marrow suppression resulting in agranulocytosis.

**Conclusion:**

Thyroid storm is a rare condition that carries a high risk of mortality and can further compromise a patient’s immune system due to complications of common treatment modalities. It can potentially be misdiagnosed as sepsis due to tachycardia, febrile state, and tachypnea. This case report includes a discussion of diagnostic studies, as well as medical and surgical treatment modalities that led to the patient’s recovery.

CPC-EM Capsule What do we already know about this clinical entity?

*Thyroid storm and agranulocytosis have high mortality. Agranulocytosis is a known side effect of hypothyroid medications, but the incidence is low.*

What makes this presentation of disease reportable?

*The clinical presentation of combined drug-induced agranulocytosis and thyroid storm is extremely rare.*

What is the major learning point?

*Agranulocytosis is a rare side effect of commonly prescribed hypothyroid medications and should be considered in settings of thyroid storm.*

How might this improve emergency medicine practice?

*Sharing rare clinical presentations provides a collective learning environment for emergency clinicians.*

## INTRODUCTION

Thyroid storm (TS) is a dangerous, life-threatening metabolic disorder that can present unique challenges in management for an emergency physician. While the incidence of TS among hospitalized patients is estimated to be low (1–2%), it carries a 12-fold increase in mortality compared to thyrotoxicosis that ranges from 8–22%.^1^ ^–^ ^3^ The use of thionamides, such as methimazole, for hyperthyroidism is common in the United States. A recent study found 60% of clinicians prefer thionamide monotherapy as the first-line treatment for hyperthyroidism.^4^ Although a mainstay of hyperthyroid management, thionamides can also be associated with agranulocytosis, a rare but dangerous side effect.^5^ Agranulocytosis can be life-threatening and is associated with a fatality rate as high as 7%.^6^

We present a case of TS in a young, otherwise healthy patient who presented to an emergency department (ED) in a large, tertiary-care hospital and was found to have concurrent agranulocytosis as a side effect of his underlying thionamide treatment. There are few reported cases describing management of TS in the setting of agranulocytosis. Special considerations should be made surrounding proper management of both coexisting conditions.

## CASE REPORT

A 26-year-old male with a past medical history of hyperthyroidism and thyromegaly presented to the ED with complaints of sore throat, fever, and diarrhea. He stated that nine days prior he began to have a progressively worsening sore throat, which prevented him from taking his prescribed methimazole (10 milligrams [mg] twice daily). Following the onset of his sore throat, he reported watery stools that occurred several times per day. He also complained of lesions to his chest, back, and oral mucosa that would appear periodically. The patient described three similar episodes over the prior year, which prompted visits to other hospitals, with the most recent episode 2–3 months earlier. He was told on his most recent visit that he would require a tonsillectomy and was discharged with outpatient follow-up.

Initial physical examination revealed an uncomfortable appearing male, visibly diaphoretic and lethargic, but oriented to person, place, and time. Vital signs were notable for a heart rate of 167 beats per minute (bpm), blood pressure of 146/70 millimeters of mercury, respiratory rate of 20 breaths per minute, oxygen saturation of 99% on room air, and a temperature of 38.6°C. He was found to have posterior oropharyngeal erythema and tonsillar edema without exudates. No goiter or thyroid masses were palpable. He was tachypneic with clear breath sounds and tachycardic with a regular rhythm and no appreciable murmurs. Skin examination was notable for multiple acneiform lesions at various stages of healing to the anterior and posterior neck and trunk.

We considered a broad differential, but our preliminary investigation favored TS as the primary diagnosis. Thyroid storm with concomitant infection was also a consideration. Diagnostic tests ordered included a complete blood count (CBC), complete metabolic panel (CMP), electrocardiogram, blood cultures, urinalysis, strep A polymerase chain reaction (PCR), severe acute respiratory syndrome coronavirus 2 (SARs-CoV-2) PCR, lactate, thyroid stimulating hormone (TSH), free triiodothyronine (T3) and free thyroxine (T4), prothrombin time (PT), and international normalized ratio (INR). We calculated the Burch-Wartofsky point scale for thyrotoxicosis with a score of 60 (15 for temperature, 10 for agitation, 10 for diarrhea, 25 for heart rate), further supporting our leading diagnosis of TS.^7^

Initial therapies ordered included propranolol 1 mg intravenously (IV), sodium chloride 0.9% one liter (L) IV, acetaminophen 650 mg orally (PO), and methimazole 20 mg PO. Shortly after initial orders were placed, the laboratory called regarding a critical CBC result, noting a white blood cell count of 0.5 × 10^9^ cells per L (reference range 4.5–11.0 × 10^9^/L). This raised concern for drug-induced agranulocytosis in the setting of TS. Upon receiving this information, we canceled the order for methimazole, and the patient was placed in a negative pressure isolation room. Broad spectrum antibiotics, including piperacillin-tazobactam and vancomycin, were administered given agranulocytosis and neutropenic fever. Additional studies were notable for a lactate of 5 millimoles (mmol) per L (0.5–2.2 mmol/L); TSH less than 0.015 international units per milliliter (IU/mL) (0.465–4.680 IU/mL); T4 greater than 6.99 nanograms per deciliter (ng/dL) (0.78–2.19 ng/dL); T3 greater than 22.80 ng/dL (2.77–5.27 ng/dL); PT 28.2 seconds (25.1–36.5 seconds); and INR 2.5 (0.9–1.1). Pertinent CMP findings included a sodium level of 130 milliequivalents (mEq) per L (135–145 mEq/L) and potassium level of 3.3 mEq/L (3.5–5.1 mEq/L).

The medical intensive care unit (MICU) was consulted for admission due to our diagnosis of TS and concomitant agranulocytosis with possible sepsis secondary to underlying bacterial illness. Clinical improvement was noted with the initial order of IV fluids and IV propranolol, including improvement of his tachycardia (110 bpm vs 167 bpm on arrival). Upon admission, the MICU ordered additional IV fluids, propranolol 40 mg PO, potassium chloride 10 mEq IV, magnesium 1g IV, and hydrocortisone sodium succinate 100 mg IV. While in the MICU the patient continued to receive beta-blockers (propranolol 60 mg PO four times daily), steroids (hydrocortisone 60 mg IV four times daily), and bile acid sequestrants (cholestyramine 4 g packet once daily). He continued to receive empiric antibiotics (piperacillin/tazobactam 4.5 g every eight hours, vancomycin 1.5 g every eight hours). He received a bone marrow biopsy, which was suggestive of methimazole-induced bone marrow suppression.

Both the endocrinology and otorhinolaryngology services evaluated the patient and determined that a thyroidectomy was required. He was subsequently transferred to another facility to have this procedure performed. Fourteen days following his initial ED presentation, he underwent a total thyroidectomy. He continued to improve clinically following the procedure and was discharged the next day with instructions regarding outpatient endocrinology follow-up.

## DISCUSSION

The recognition and treatment of thyroid emergencies is well within the scope of practice for the emergency physician. Isolated TS can be treated with a stepwise approach that has been well documented in the literature. Standard first-line pharmacotherapy in TS aims to block production and release of thyroid hormones with propylthiouracil or methimazole, inhibit release of preformed thyroid hormones with iodine, decrease peripheral conversion of T4 to T3 with propylthiouracil and steroids, and treat adrenergic symptoms with beta-blockade, prior to definitive treatment in the form of surgery or radioactive iodine ablation.^8^

Our patient represents a rare and complex case of TS with concurrent methimazole-induced agranulocytosis and neutropenic fever, who met sepsis parameters with symptoms that raised concern for underlying infectious process. This case highlights multiple challenges surrounding conventional TS treatment when standard, first-line pharmacotherapy also contributes to an alternate, life-threatening condition. To our knowledge, no specific literature or guidelines exist regarding the management of this presentation.

Given the inherently high mortality rate of both neutropenic sepsis and TS, it was imperative to implement therapeutic measures to optimally treat both conditions simultaneously, while also minimizing iatrogenic risk to the patient. It is well known that sepsis can mimic features of TS, and given that our patient presented with concurrent drug-induced agranulocytosis along with infectious symptomatology and concerning physical examination features, we could not exclude an underlying bacterial infectious process.^9^ Drug-induced agranulocytosis occurs in less than seven cases per million individuals per year.^10^ Most research regarding agranulocytosis is associated with bone marrow suppression in cancer treatment, and neutropenic fever has been identified as an important criterion in determining life-threatening infections in this specific patient population.

Neutropenic fever is defined as absolute neutrophil count less than 500 cells per microliter and fever as a single oral temperature of greater than 38.3°C or greater than 38.0°C sustained over a one-hour period.^11^ Furthermore, high-risk patients with neutropenic fever are those with any of the following: hemodynamic instability; oral or gastrointestinal mucositis; gastrointestinal symptoms including abdominal pain, nausea, vomiting, or diarrhea; neurologic or mental-status changes; intravascular catheter infection; new pulmonary infiltrate; or hypoxemia. Neutropenic sepsis has been shown to approach mortality of greater than 40% if left untreated at 48 hours.^12^ Given our patient’s underlying neutropenia, fever, and increased risk for infection, we made the decision to treat for neutropenic fever and sepsis immediately with IV fluid resuscitation and broad-spectrum IV antibiotics.

The patient’s underlying agranulocytosis and neutropenia conflicted with the standard treatment options intended to control thyroid hormone production. Controlling the production of new thyroid hormone is imperative in the setting of TS by using thionamides; however, the first step in treating drug-induced agranulocytosis is to also stop the offending agent. Additionally, without a thionamide on board, it is contraindicated to give potassium iodide or Lugol’s iodine in the ED, as it can increase thyroid hormone release.

Following the conventional stepwise approach for TS treatment, high-dose IV steroids were also considered; however, the use of high-dose steroids is associated with increased mortality in septic patients without neutropenia.^8^ ^,^ ^13^ In patients with neutropenic sepsis, hydrocortisone therapy has been associated with increased adverse events and has not been shown to improve mortality.^14^

Standard treatment of TS also requires the use of IV beta-blockers, which decrease sympathetic activity during TS and prevent peripheral conversion of T3 to T4. Use of beta-blockers in our patient required special scrutiny given his neutropenic fever and concern for concomitant sepsis from a bacterial source. Although it is controversial, recent literature suggests that beta-blocker therapy may be safe in septic patients.^15^ We decided the therapeutic benefits of beta-blockers in TS outweighed the risks of beta-blockade in the setting of potential sepsis, given that our patient was otherwise healthy and remained hemodynamically stable during his course of care.

Ultimately while under the care of the ED team, the patient was treated with IV beta-blockers, acetaminophen, IV fluids, and broad-spectrum antibiotics. His clinical status improved significantly following these measures, and he eventually required a total thyroidectomy as curative therapy.

## CONCLUSION

Agranulocytosis, sepsis, and thyroid storm are life-threatening presentations that can be associated with hyperthyroidism. Together they present a unique diagnostic and therapeutic challenge for the emergency physician treating the thyroid-toxic patient, especially when sepsis is an additional consideration. Deviations from standard TS treatment guidelines should be considered in this patient population to avoid any potential complications and harm to them.

## References

[1] Klubo-Gwiezdzinska J Wartofsky L. Thyroid emergencies. Med Clin North Am. 2012;96(2):385–403. 22443982 10.1016/j.mcna.2012.01.015

[2] Angell TE Lechner MG Nguyen CT et al. Clinical features and hospital outcomes in thyroid storm: a retrospective cohort study. J Clin Endocrinol Metab. 2015;100(2):451–9. 25343237 10.1210/jc.2014-2850

[3] Swee du S Chng CL Lim A. Clinical characteristics and outcome of thyroid storm: a case series and review of neuropsychiatric derangements in thyrotoxicosis. Endocr Pract. 2015;21(2):182–9. 25370315 10.4158/EP14023.OR

[4] Brito JP Payne S Singh Ospina N et al. Patterns of use, efficacy, and safety of treatment options for patients with Graves’ disease: a nationwide population-based study Thyroid. 2020;30(3):357–64. 31973681 10.1089/thy.2019.0132

[5] van Staa TP Boulton F Cooper C et al. Neutropenia and agranulocytosis in England and Wales: incidence and risk factors. Am J Hematol. 2003;72(4):248–54. 12666135 10.1002/ajh.10295

[6] Ibáñez L Vidal X Ballarín E et al. Population-based drug-induced agranulocytosis. Arch Intern Med. 2005;165(8):869–74. 15851637 10.1001/archinte.165.8.869

[7] Burch HB Wartofsky L. Life-threatening thyrotoxicosis. Thyroid storm. Endocrinol Metab Clin North Am. 1993;22(2):263–77. 8325286

[8] Tintinalli JE. Endocrine disorders. In: Tintinalli’s Emergency Medicine: A Comprehensive Study Guide, 9th ed. (p. 1454). New York, NY: McGraw-Hill Education; 2019.

[9] Rayner SG Hosseini F Adedipe AA. Sepsis mimicking thyroid storm in a patient with methimazole-induced agranulocytosis. BMJ Case Reports. 2013;2013(jul16 1):bcr2013200145-b. 10.1136/bcr-2013-200145 PMC3736612 23861276

[10] Strom BL Carson JL Schinnar R et al. Descriptive epidemiology of agranulocytosis. Arch Intern Med. 1992;152(7):1475–80. 1627027

[11] Freifeld AG Bow EJ Sepkowitz KA et al. Clinical practice guideline for the use of antimicrobial agents in neutropenic patients with cancer: 2010 update by the Infectious Diseases Society of America. Clin Infect Dis. 2011;52(4):e56–93. 21258094 10.1093/cid/cir073

[12] Bodey GP Jadeja L Elting L. Pseudomonas bacteremia. Retrospective analysis of 410 episodes. Arch Intern Med. 1985;145(9):1621–9. 3927867 10.1001/archinte.145.9.1621

[13] Penack O Becker C Buchheidt D et al. Management of sepsis in neutropenic patients: 2014 updated guidelines from the Infectious Diseases Working Party of the German Society of Hematology and Medical Oncology (AGIHO). Ann Hematol. 2014;93(7):1083–95. 24777705 10.1007/s00277-014-2086-0 PMC4050292

[14] Kang J Han M Hong SB et al. Effect of adjunctive corticosteroid on 28-day mortality in neutropenic patients with septic shock. Ann Hematol. 2019;98(10):2311–8. 31432214 10.1007/s00277-019-03785-w

[15] Hasegawa D Sato R Prasitlumkum N et al. Effect of ultrashort-acting β-blockers on mortality in patients with sepsis with persistent tachycardia despite initial resuscitation: a systematic review and meta-analysis of randomized controlled trials. Chest. 2021;159(6):2289–300. 33434497 10.1016/j.chest.2021.01.009

